# Primary care capitation payments in the UK. An observational study

**DOI:** 10.1186/1472-6963-10-156

**Published:** 2010-06-08

**Authors:** Gwion Rhys, Hendrik J Beerstecher, Claire L Morgan

**Affiliations:** 1 Ty Doctor, Ffordd Dewi Sant, Nefyn, LL53 6EA, UK; 2 Canterbury Road Surgery, 111 Canterbury Road, Sittingbourne, Kent, ME10 4JA, UK

## Abstract

**Background:**

In 2004 an allocation formula for primary care services was introduced in England and Wales so practices would receive equitable pay. Modifications were made to this formula to enable local health authorities to pay practices.

Similar pay formulae were introduced in Scotland and Northern Ireland, but these are unique to the country and therefore could not be included in this study.

**Objective:**

To examine the extent to which the Global Sum, and modifications to the original formula, determine practice funding.

**Methods:**

The allocation formula determines basic practice income, the Global Sum. We compared practice Global Sum entitlements using the original and the modified allocation formula calculations.

Practices receive an income supplement if Global Sum payments were below historic income in 2004. We examined current overall funding levels to estimate what the effect will be when the income supplements are removed.

**Results:**

Virtually every Welsh and English practice (97%) received income supplements in 2004. Without the modifications to the formula only 72% of Welsh practices would have needed supplements. No appreciable change would have occurred in England.

The formula modifications increased the Global Sum for 99.5% of English practices, while it reduced entitlement for every Welsh practice.

In 2008 Welsh practices received approximately £6.15 (9%) less funding per patient per year than an identical English practice. This deficit will increase to 11.2% when the Minimum Practice Income Guarantee is abolished.

**Conclusions:**

Identical practices in different UK countries do not receive equitable pay. The pay method disadvantages Wales where the population is older and has higher health needs.

## Background

The General Medical Services (GMS) contract for delivery of primary care services has existed since the inception of the UK National Health Service (NHS) in 1948. For most of the period, primary care practices received a complex mixture of capitation fees, reimbursements for expenses, fee for services, and a small amount of pay for performance. In 2004, the funding of primary care practices was radically modified as part of a new GMS contract.

Each practice now receives three key income streams. The majority of practice funding comes from protected capitation fees -- the minimum practice income guarantee (MPIG). Additional income is derived from the Quality and Outcomes Framework as performance related pay, and supplementary (enhanced) services, which are mostly paid on fee for service basis [1].

Pay-for-performance and fee-for-service are fairly straightforward, but the MPIG and the methodology of its calculation are less well understood. One of the aims of the new GMS contract was to achieve equitable pay for basic service provision, to "recognise casemix and practice circumstances, and ensure money will flow according to patient need" [2]. For this purpose the Carr-Hill allocation formula was introduced to determine the Global Sum, which represents core funding.

The proposed core funding was lower than existing income for most practices and there was a risk that physicians would reject the proposed changes. To ensure acceptance of the proposals a practice's 2003 funding was compared to the Global Sum and any deficit was made good by a "Correction Factor". As a consequence core funding became dependent on historical funding patterns making the allocation formula financially irrelevant.

In 2005 the government indicated that the Correction Factor would be phased out, something also suggested by the National Audit Office [3-5]. The future financial stability of a practice will therefore become dependent on the Carr-Hill allocation formula.

In this report we assess the impact of the proposed changes on practices, and how two modifications to the original Carr-Hill formula affect individual practices.

## Methods

### Core funding

Although the new payment methods applied to the UK as a whole, some functions like the funding of health care are devolved to the constituent nations: England, Wales, Scotland and Northern Ireland. At a lower level, the national governments have decentralised GMS contracts, which are now administered by local health authorities. This meant that even centrally determined payments have to be administered by local health authorities.

This necessitated a complex hierarchy for core funding payments. All data for the determination of practice core funding are held centrally. However, local health authorities are required to repeat the calculations for the locally contracted practices. Central government provides a key, the 'PCO Weighted Listsize Normalising Index', to local health authorities to effect a national correction to the local data.

### Ethical approval

This report uses publicly available data that does not relate to individuals and the Kent ethical committee confirmed no approval was required for this project.

### Control and study groups

Our control group comprised all English (n = 8436) and Welsh (n = 494) practices. For these practices, data were obtained on practice list size (the number of patients registered with the practice) and socio-economic deprivation. We also received the centrally held formula indices, which determine the weighted practice list (April 2007).

Local health authorities are called Primary Care Trust (PCT) in England and Local Health Board (LHB) in Wales. We refer to them under the common term Primary Care Organisation (PCO).

To compare formula payments to actual practice income we requested practice supplemental payments (Correction Factors). Our study group comprises 882 English and 486 Welsh GMS practices in 25 and 22 PCOs respectively. The Correction Factors were supplied for 100% of the Welsh practices, and for 680 practices in 19 of the 25 randomly selected English PCOs (76%). Practice Correction Factor payments were identical between 2004 and 2009.

The 19 acquiescing English PCOs were not statistically different from the 25 randomly selected PCOs we approached in respect of practice size, socio-economic deprivation, allocation formula indices, weighted practice list and formula determined practice income.

### Carr-Hill allocation formula and Global Sum calculation

The Carr-Hill allocation formula was designed to ensure equity of primary care practice funding. The formula uses six indices to determine patient weightings: age-sex; additional needs; list turnover; practice market forces factor; rurality; and count of patients in nursing/residential homes (Table 1).

**Table 1:** Indices used in allocation formula

Index	Brief description of index	Interquartile range^†^	Change in percentage explained^‡^
Age Sex Index	Compensates for higher consulting rates for women and patients at the extremes of age by weighting patients according to age and sex	0.93 - 1.05	39%
Additional Needs Index	Deprivation related clinical workload indicator generated using Standard Mortality and Long Standing Illness data	0.91 - 1.10	58%
Rurality Index	Generated from population density data and average distance of patients' homes from the practice main surgery	0.97 - 1.01	11%
MFF* Index	Represents local labour costs and calculated according to the practice main surgery address	0.97 - 1.02	7%
List Turnover Index	Compensates for high patient turnover and generated from counts of new patients	0.99 - 1.01	3%
NRH* Index	Compensates for additional home visits generated by patients in nursing and residential care	1.00 - 1.00	3%

† Original formula data for English and Welsh practices (1-4-2007).

‡ Change in percentage explained if index is removed from a multiple regression model containing all indices as predictors for overall Carr-Hill formula weighting (original formula data 2007). The model with all indices explains 97% of the variation of Carr-Hill weighting.

* MFF denotes Market Forces Factor and NRH Nursing and Residential Home patients.

Each index is calculated in a unique way. For instance the age-sex index assumes the work generated by one female aged 75 to 84 is equal to 6.56 males aged 5 to 14 [6]. A patient that joins the practice is counted as 1.46 patients for the first year after registration [6]. The rurality index is a function of the distance the patient lives from the surgery and the population density; its value based on the expenses of a sample of primary care physicians [6].

These indices, all centred around 1, are multiplied by each other to give an overall practice weighting [6]. A perfectly average practice would have six indices of 1, which means the normalised weighted practice population is identical to the 'raw' practice population. The normalised weighted practice population is multiplied by £54.72 (2004 to 2009) to yield the Global Sum. For instance a practice with 1,000 patients and a formula weighting of 0.9 and would receive 900 × 54.72 = £ 49,248 per annum in Global Sum funding. Age profile and the morbidity/mortality index are the strongest predictors of practice Global Sum (Table 1).

Two methods of calculating the relative need of practices were published [6,7]. The original publication inferred that an individual practice was compared to all other practices in England and Wales. However a later publication introduced PCO elements into the indices, which relate local practices to each other [8]. The key to unlock the modified method is the "PCO weighted list size normalising index", determined centrally and based on previous rather than current quarter data.

The example below demonstrates how the original and modified methods work. For details see additional file 1.

### Example: calculating the Age-Sex index for the original and modified method

Original age sex index (ASI_E+W_) =

Modified age sex index (ASI_loc_)^† ^=

^† ^This formula was simplified from the published one: Please refer to additional file 1 for the reworking process.

### Original and modified formulae

We calculated core funding with the original formula, which looks at practice need relative to all other English and Welsh practices. The modified formula compares individual practice weighting with neighbouring practices in the local PCO in the same home country (England or Wales) (Additional file 2).

The complex calculations obscure two simple differences between the modified and original formulae: (1) normalisation at national instead of union level and (2) the use of previous quarter instead of current quarter data.

### Minimum Practice Income Guarantee (MPIG)

Using the Global Sum calculations we determined the MPIG practice pay on 1 April 2007 for our study group practices as follows:

### Socio-economic deprivation

We used the Additional Needs Index (ANI) as a measure of socio-economic deprivation. A more conventional measure is the Index of Multiple Deprivation, but this is not available at practice level for Wales.

The ANI is closely related to the patient-postcode Index of Multiple Deprivation (IMD) in England (r = 0.93) [9]. Both ANI and IMD are based on patient postcodes. Most studies to date have used practice postcode IMD. However practice postcode IMD is less strongly related to patient postcode IMD (r = 0.81) than ANI and although ANI is not ideal, it seems at least as accurate as the more conventionally used practice postcode IMD [10].

The accuracy of using deprivation indices based on small areas has been questioned, however this is the most accurate indicator currently available [8,11].

### Statistical methods

We used Stata v.7 and Microsoft Excel^® ^for statistical analyses. T-tests were used to compare practice demographics and payments. To give an indication of the relative importance of each index, the 2007 original formula data were used to build a multiple regression model with all six indices as predictors for Carr-Hill formula weighting. Six further models were constructed by removing each index in turn. We then used the difference in adjusted R^2 ^to give an indication of the relative importance of each index.

## Results

### Original and modified formulae compared

Global Sum payments for Welsh practices based on the modified formula were 10.8-12.2% lower than those based on the original formula. There were no such striking discrepancies for English practices, where the average difference between the modified and original formula payments was 0.6%.

We find no significant Global Sum funding difference between English and Welsh practices using the modified formula (£0.21 higher in Wales, p = 0.48). However using the original formula without national normalisation, the average Global Sum for Welsh practices is £6.80 higher per patient (p < 0.0001) (Table 2 and additional file 3). The average Welsh practice looks after a more deprived and elderly population in a more remote setting (Table 3).

**Table 2:** Average payments and characteristics of study and control groups

	All Welsh practices (n = 494)	All English practices (n = 8436)	Study group Welsh practices (n = 486)	Study group English practices (n = 680)
Modified Global Sum, *a*	£ 55.26	£ 55.05	£ 55.29	£ 55.94
Original Global Sum, *b*	£ 61.50	£ 54.70	£ 61.53	£ 54.61
Total Funding MPIG, *c*	-	-	£ 68.08	£ 67.31
				
Adjusting effect (*a-b*)	£ -6.24	£ 0.35	£ -6.24	£ 0.33
Income support (CF) (*c-a*)	-	-	£ 12.79	£ 12.37
Net income support (*c-b*)	-	-	£ 6.56	£ 12.70
				
Additional Needs Index	1.08	1.01	1.08	0.96
Average List Size	6293	6377	6344	6456
Modified needs weighting	1.01	1.01	1.01	1.00
Original needs weighting	1.12	1.00	1.12	1.00

All funding figures are expressed in £ per patient per year

Global Sum = Weighted-normalised list × £54.72

MPIG = Minimum Practice Income Guarantee = Modified Global Sum + Correction Factor

CF = Correction Factor

Original Needs Weighting = number of weighted patients/number of patients (using original formula)

Modified Needs Weighting = number of weighted patients/number of patients (using modified formula)

Details of t-tests comparing the various groups are listed in additional file 3

**Table 3:** Average national value of indices calculated with the original formula (English and Welsh control groups)

	Age Sex Index	Additional Needs Index	Rurality Index	Market Forces Factor	List turnover index	Nursing and Residential home index
Wales	1.03	1.08	1.04	0.98	0.99	1.00
England	0.99	1.01	0.99	1.00	1.00	1.00
						
*t*	8.05	12.82	17.45	-12.14	-8.79	0.28
p	<0.001	<0.001	<0.001	<0.001	<0.001	0.78

The original and modified formulae differed in two aspects: Use of previous quarter data and normalisation at national rather than union level [12]. To determine the contribution of each modification we calculated payments using current quarter instead of previous quarter data. All but one of the 8,930 Welsh and English practices experienced less than 1% difference in pay through the use of previous quarter data indicating that virtually all differences between original and modified Global Sum payments were caused by the process of normalisation at national level.

There are 494 Welsh and 8,436 English practices. The formula modifications transfer approximately £19 Million Global Sum funding from Wales to England, which represents a gain of £2,252 for the average English practice, but a loss of £38,641 for the average Welsh practice. (Table 2: adjusting effect × average list × number of practices in nation).

### MPIG income supplement

In our study group, only 12 of the 464 (2.6%) Welsh practices received sufficient Global Sum in 2004 to negate the need for income supplements. The 2004 data could not be supplied by Connecting for Health and we used 2007 data to estimate how many practices would have needed MPIG income supplements with the original formula.

The original Global Sum exceeded historic income for 130 of the 464 Welsh practices (28%), and these practices would not have needed MPIG income supplements. In our English study group 21 of 680 (3.1%) practices receive no income supplements, this did not change using the original instead of the modified formula.

### Practice characteristics

We compared our study and control groups for average raw practice list, original and modified Global Sum funding, ANI, MPIG and Correction Factor.

The study group in Wales contained 98% of all Welsh practices and was therefore representative for Welsh practices in all aspects. The English study group was representative in all aspects except for deprivation related workload (ANI).

There were no differences between average English and Welsh practice list sizes or modified Global Sum, but English and Welsh groups differed in all other aspects. Details of t-tests comparing the nations are given in Table 3 and additional file 3.

### Physician income

The information commissioner for the NHS reports on primary care physician income each year. The 2007/08 report indicated that the income of GMS practitioners in Wales was £93,366 and in England £104,898 [13]. There were 1,759 Welsh GMS contractors with 3,108,809 patients and 15,144 English GMS contractors with 29,150,941 patients in 2007. However, after applying the formula to assign weightings to patients based on the six workload indicators, there were 3,458,728 weighted Welsh and 28,992,823 weighted English patients.

## Discussion

A pay formula determines core practice funding for General Medical Services (GMS) practices since 2004. The allocation formula is based on consultation patterns, which correspond to practice workload and therefore the implied use of resources.

The formula uses practice demographics like differences in age, sex, deprivation related workload and population density to assign weightings to patients. Further refinements are made to compensate for patients in residential institutions, newly registered patients and fixed costs, like staff pay, that can differ by locality.

In 2006 most English Primary Care Organisations (PCOs) were reorganised trough mergers. The mergers unexpectedly changed our formula indices. During an exercise to verify the accuracy of the indices we found persisting small (<1%) discrepancies affecting English practices. Using the raw data and the original formula, we recalculated the indices and found additional large (10.8% to12.2%) discrepancies exclusively involving Welsh practices.

Before 2004, GMS contracts were between the secretary of state of the central government and the individual primary care physician. The new GMS contracts are between the practice and local PCOs. The change required routing payments from central budgets through the complex hierarchy of union, home countries (nations) and health authorities (PCOs). Modifications were made to the allocation formula, introducing regional and national adjustments. The modifications to the formula are responsible for the discrepancies in practice funding.

Normalisation or equalisation of the populations at national level means that the higher age profile and socio-economic deprivation of Wales is no longer taken into account. At practice level this means the average Welsh practice receives £6.80 (11.2%) less Global Sum funding per patient per annum, compared to an identical practice in England.

However, for virtually all practices (97%) with a GMS contact, core funding does not depend on the formula, it depends on historic funding. Historic funding was perpetuated through supplementary payments by the income protection scheme since 2004. The 11.2% lower Global Sum for Welsh practices could be academic if it was negated by higher supplemental payments from the income protection scheme.

To estimate the actual funding of Welsh practices we sampled supplementary payments. There was no significant difference in supplementary payments between Wales and England, £12.79 and £12.38 respectively. According to the original formula, the annual capitation value of Welsh patients is on average £6.80 (11.2%) higher than for English patients. In our study groups the formula values Welsh patients £6.92 higher than English patients. This figure represents differences in caseload-adjusted funding to compensate for increased pressure on resources. However, instead of receiving £6.92 more, Welsh practices only receive on average £0.77 extra funding, £0.35 of this in Global Sum and £0.42 more income protection. If we generalise the finding in our study group to the national figures, then Welsh practices received historically approximately £6.15 (9% of £68.08) less per patient per year than the formula affords to an identical English practice.

In 2004 and 2005 normalising was performed annually. Over the course of the year identical practices received diverging payments simply because they were located in different regions [3]. In 2006 this inequity was addressed in England by calculating the Global Sum entitlements quarterly instead of annually. This way, the inequities build up for a quarter instead of a year and pay adjustments are less noticeable to practices.

However, the problem of national inequities of practice income was never acknowledged. By normalising payments nationally, the higher Welsh case-mix burden is scaled back; therefore a practice in Wales receives lower Global Sum funding than an identical practice located in England.

The introduction of the formula has uncovered a relative historic funding deficit for Welsh practices. Although practices in England and Wales historically received similar pay per patient, the allocation formula revealed pay was inequitable when workload factors were taken into account. Phasing out the income protection without correcting the inequity of normalisation at national level means Welsh practices will lose the small ameliorating effect of the MPIG and the relative income deficit will grow from 9% to 11.2%.

If we accept that the formula correctly predicts the use of resources, then it would be expected that Welsh GPs work 11.2% harder or earn 11.2% less NHS income. There is some indication that this may be the case in the figures published by the Information Commissioner. In 2007, the technical steering committee reported that GMS contractors earned on average 11% less in Wales [13]. The average number of patients per GMS contractor is 8% lower in Wales (1,767 versus 1,925), leaving an apparent unadjusted 3% income deficit for Welsh practices.

However, if we accept that the formula correctly adjusts for workload, then the case-mix corrected number of patients per contractor is 3% higher in Wales: 1,966 against 1,914 in England. If we accept that the formula distributes resources fairly, then Welsh primary care physicians should earn 3% more instead of 11% less than their English counterparts, representing a 14% case-mix adjusted income deficit.

### Strengths and weaknesses

The English study group represents a smaller proportion of practices (680 of 8436, 8%) than our Welsh study group (486 of 494, 98%). Whilst correction factor data were supplied by all Welsh LHBs, 76% of English PCTs provided the same information.

The English study group of 25 randomly selected PCTs is representative for average English practice size and Global Sum income. However, there is a difference between the deprivation-related workload of our study group and the English average. It is possible, owing to the small differences in deprivation-related workload, that MPIG income support was systematically higher or lower in the English study group, therefore the MPIG calculations for our population may not be representative for England, and must be taken as an estimate. This problem does not affect the Global Sum calculations or this paper's conclusions, which are based on whole populations of both nations.

Similar inequities in Global Sum income may exist in Scotland and Northern Ireland, the other two home countries (nations) in the United Kingdom. It should be possible to express average national need by comparing total weighted patients. However, that would require the same raw data to be available at practice level, which does not currently exist as far as we could ascertain. Additionally, while the English and Welsh indices can be derived from raw demographic data, the Scottish method for calculating formula pay combines the demographic data for a region before assigning this to a patient, meaning that raw index data do not exist at practice level. Considerable funding would be needed to create the data to investigate national inequities in the other nations of the United Kingdom.

The funding disparities did not enable us to determine the validity of the Carr-Hill funding formula. Although the allocation formula has been introduced and the income guarantee is being phased out, the formula design has potential shortcomings [14]. The architects of the formula indicated in earlier papers that the formula might be too volatile to be used at practice level [15].

Ideally a needs based formula is preferred over a utilisation based formula. Utilisation formulae could perpetuate historic spending patterns instead of redistributing funding to areas with the highest need [16]. The Carr-Hill allocation formula was developed pragmatically, by measuring how long the electronic health record (EHR) was accessed and this was compared to a variety of factors, like age, gender and morbidity/mortality [8,17]. Staff were categorised and nominal pay grade assigned to put a monetary value on the episode. This means the formula is essentially utilisation and not needs based. Expressing 'work' as EHR usage also opens the possibility for patient or practice factors to skew funding.

Furthermore, the existence of different formulae casts doubt on the intrinsic validity of the indices or the databases on which they are based. For instance, the Scottish allocation formula has a different age-sex weighting and the formula review group proposed yet another weighting, based on a third database [8,18].

We did not find any articles discussing the inequities uncovered in this report. UK healthcare funding is centrally determined and political rather than scientific reasoning seems to drive changes to primary care funding. It could be argued that different or flexible funding methods encourage innovation or fund increased capacity, like growth monies intended for Personal Medical Services (PMS) contracts [19-22]. However, it would seem immoral to offer different payments to various contractors providing identical services. In GMS, the Carr-Hill formula determines the Global Sum to fund Essential Services: Consultation, treatment and referral of patients [23]. The funding disparities uncovered in this report are not linked to innovation or additional capacity. Flexibilities for GMS contractors are funded separately through additional services, enhanced services and the quality scheme.

Formula determined funding seems a morally defensible way to pay various contractors for identical services. If pay formulae are sufficiently reliable, logic would demand their ubiquitous application. If they are not, the question is whether they should be used at all.

## Conclusions

The objective of introducing the allocation formula was to ensure equity, so that practices with the same clinical workload would receive the same, case-mix corrected, payments. This strategy has failed as national normalisation causes pay inequities of 10.8% to 12.2% at practice level.

## Competing interests

GR is a GMS contractor in Wales and HB is a GMS contractor in England. Both are affected by changes in GMS funding. The GMS contract is negotiated nationally and this report will not directly affect either author.

No funding was received by the authors for this study or any activities associated with this study.

## Authors' contributions

The authors jointly designed the study, collected and analysed the data and jointly drafted the manuscript. All authors read and approved the final manuscript.

## Pre-publication history

The pre-publication history for this paper can be accessed here:

http://www.biomedcentral.com/1472-6963/10/156/prepub

## Supplementary Material

Additional file 1 **Reworking of the local age sex index**. Click here for file

Additional file 2 **Calculating payments using the original and the modified methods**. Click here for file

Additional file 3 **T-tests for the various practice characteristics in Wales and England**. Click here for file
