# Supplementary material for: Primary care capitation payments in the UK. An observational study
Source: BMC Health Serv Res. 2010 Jun 8;10:156. doi: 10.1186/1472-6963-10-156 (PMC2889945; doi:10.1186/1472-6963-10-156)
Supplement: Additional file 1 — Reworking of the local age sex index. [file 1472-6963-10-156-S1.DOC]

**Additional file 1: Reworking of the local age sex index:**

The guidance for the modified Global Sum calculations uses a formula that in itself consists of formulae. For clarity we reworked the formula in the main text of the article and this box is to show how this was done starting with the published formula for the age-sex index [7]:

$$\frac{practice age sex weighted list}{practice normalised List}\times\frac{PCO weighted Population CQ}{PCO age sex weighted list}$$

PCO weighted Population CQ (Current Quarter) = PCO Raw Population in the current quarter x PCO Weighted Listsize Normalising Index

Practice normalised list = Practice List Size x (PCO Weighted Population CQ / PCO Raw Population Curr Qtr)

Substituting these definitions in the Department of Health local index yields the local age sex index used as example in body text.:

=

$$\frac{practice age sex weighted list\times PCO raw list\times PCO weighted listsize normalis ing index\times PCO raw list CQ}{practice raw List\times PCO raw list CQ\times PCO weighted listsize normalis ing index\times PCO age sex weighted list}$$

=

$$\frac{practice age sex weighted list}{practice raw List}\times\frac{PCO raw list}{PCO age sex weighted list}$$
