# Supplementary material for: Primary care capitation payments in the UK. An observational study
Source: BMC Health Serv Res. 2010 Jun 8;10:156. doi: 10.1186/1472-6963-10-156 (PMC2889945; doi:10.1186/1472-6963-10-156)
Supplement: Additional file 2 — Calculating payments using the original and the modified methods. [file 1472-6963-10-156-S2.DOC]

**Additional file 2: Calculating payments using the original and the modified methods:**

**Original method:** the six indices based on the total population are multiplied with each other and with the practice list, and finally with £54.72. Normalisation is to the total population (England + Wales) rather than to the national or local population.

$$\left[ASI\right._{E+W}\times ANI_{E+W}\times LTI_{E+W}\times MMF_{E+W}\times RI_{E+W}\times\left.NHI_{E+W}\right]\times P ractice raw list\times£54.72$$

Multiplying all indices results in an overall weighting for the list:

=$\left[\frac{practice weighted list}{practice raw List}\right.\times\left.\frac{English+Welsh raw list}{English+Welsh weighted list}\right]\times P ractice raw list\times£54.72$

=$\frac{English+Welsh raw list}{English+Welsh weighted list}\times P ractice weighted list\times£54.72$

=$P ractice normalised weighted list (England \underline{and} Wales)\times£54.72$

**Modified method:** the six indices based on the local population are multiplied with each other, with the practice list, and divided by the PCO weighted list. This is then multiplied by the PCO weighted population and divided by the PCO weighted list and finally multiplied with £54.72. Some of the elements of the formula are in themselves formulae [7] and can therefore be re-written in several ways:

$$\frac{P ractice raw list\times PCO weighted list CQ}{PCO raw list CQ}\times\left[ASI_{loc}\right.\times ANI_{loc}\times LTI_{loc}\times MMF_{loc}\times RI_{loc}\times\left.NHI_{loc}\right]\times\frac{PCO weighted list CQ}{PCO weighted list}\times£54.72$$

**The PCO weighted list CQ = the PCO raw list x the PCO Weighted List Size Normalising Index (WLSNI)**

=$\frac{P ractice raw list\times PCO weighted list CQ}{PCO raw list CQ}\times\left[\frac{P ractice weighted patients}{P ractice raw patients}\right.\times\left.\frac{PCO raw patients}{PCO weighted patients}\right]\times\frac{PCO raw list}{PCO weighted list}\times PCO WLSNI\times£54.72$

**The PCO WLSNI = PCO normalised overall weighted patients divided by PCO total registered population.**

=$P ractice weighted patients\times\frac{PCO raw list}{PCO weighted list}\times\frac{PCO normalised weighted patients}{PCO raw patients}\times£54.72$

=$P ractice weighted patients\times\frac{PCO raw list}{PCO weighted list}\times\frac{PCO weighted patients}{PCO raw patients}\times\frac{National raw patients}{National weighted patients}\times£54.72$

=$P ractice weighted patients\times\frac{National raw patients}{National weighted patients}\times£54.72$

=$P ractice normalised weighted list (England \underline{or} Wales)\times£54.72$

Owing to the use of the previous quarter normalisation in the modified method there was a slight difference of total English+Welsh populations on 01-04-2007: 56,451,651 (UK original formula) and 56,905,089 (UK modified formula) [12]. To enable exact comparison at practice level the two methods, we equalised the outcomes of the original and modified formulae by multiplying the original formula with:

$\times\frac{\sum Normalised weighted list_{England \underline{or} Wales}}{\sum Normalised weighted list_{England \underline{and} Wales}}$ = 1.008032

ASI = Age Sex Index, ANI = Additional Needs Index, LTI = List Turnover Index, MMF = Market Forces Factor, RI = Rurality Index, NHI = Nursing Home Index, WLSNI = Weighted List Size Normalising Index.
