# Supplementary material for: Primary care capitation payments in the UK. An observational study
Source: BMC Health Serv Res. 2010 Jun 8;10:156. doi: 10.1186/1472-6963-10-156 (PMC2889945; doi:10.1186/1472-6963-10-156)
Supplement: Additional file 3 — T-tests for the various practice characteristics in Wales and England. [file 1472-6963-10-156-S3.DOC]

# Additional file 3: T-tests for the various practice characteristics in Wales and England

| **Additional Needs Index** | **Mean Value** | Welsh control group | Welsh study group | English control group | English study group |
| --- | --- | --- | --- | --- | --- |
| Welsh control group | 1.08 | - |  |  |  |
| Welsh study group | 1.08 | t = 0.08  p = 0.94 | - |  |  |
| English control group | 1.01 | t=12.82 p<0.01 | t = 12.82  p<0.01 | - |  |
| English study group | 0.96 | t=18.63  p<0.01 | t=18.82  p<0.01 | t=10.37  p<0.01 | - |

| **Practice List Size** | **Mean** Value | Welsh control group | Welsh study group | English control group | English study group |
| --- | --- | --- | --- | --- | --- |
| Welsh control group | 6293 | - |  |  |  |
| Welsh study group | 6344 | t=0.22 p=0.83 | - |  |  |
| English control group | 6378 | t=0.46 p=0.65 | t=0.18 p=0.86 | - |  |
| English study group | 6456 | t=0.76 p=0.45 | t=0.52  p=0.60 | t=0.50  p=0.62 | - |

| **Modified Global Sum funding (£)** | **Mean** Value | Welsh control group | Welsh study group | English control group | English study group |
| --- | --- | --- | --- | --- | --- |
| Welsh control group | 55.28 | - |  |  |  |
| Welsh study group | 55.29 | t=0.04  p=0.97 | - |  |  |
| English control group | 55.05 | t=0.75  p=0.46 | t=0.79  p=0.43 | - |  |
| English study group | 54.94 | t=1.06  p=0.29 | t=1.10  p=0.27 | t=0.46  p=0.64 | - |

| **Original Global Sum Funding (£)** | **Mean**  **Value** | Welsh control group | Welsh study group | English control group | English study group |
| --- | --- | --- | --- | --- | --- |
| Welsh control group | 61.51 | - |  |  |  |
| Welsh study group | 61.53 | t=0.04  p=0.97 | - |  |  |
| English control group | 54.70 | t=22.99  p<0.01 | t=22.87  p<0.01 | - |  |
| English study group | 54.61 | t=20.47  p<0.01 | t=20.49  p<0.01 | t=0.35  p=0.73 | - |

| **Correction Factor Funding (£)** | **Mean**  **Value** | Welsh study group | English study group |
| --- | --- | --- | --- |
| Welsh study group | 12.79 | - |  |
| English study group | 12.38 | t=1.99  p=0.37 | - |

| **Protected Historic MPIG Funding (£)** | **Mean**  **Value** | S Welsh MPIG | S English MPIG |
| --- | --- | --- | --- |
| Welsh study group | 68.08 | - |  |
| English study group | 67.31 | t=1.59 p=0.11 | - |

* Funding in Pounds (£) per patient per year.
